# Forskolin-Loaded Halloysite Nanotubes as Osteoconductive Additive for the Biopolymer Tissue Engineering Scaffolds

**DOI:** 10.3390/polym13223949

**Published:** 2021-11-15

**Authors:** Ekaterina Naumenko, Ivan Guryanov, Elena Zakirova, Rawil Fakhrullin

**Affiliations:** Institute of Fundamental Medicine and Biology, Kazan Federal University, Kreml Uramı 18, Kazan 420008, Republic of Tatarstan, Russian Federation; ivan.guryanov@gmail.com (I.G.); lenahamzina@yandex.ru (E.Z.)

**Keywords:** forskolin, biopolymers, tissue engineering scaffolds, halloysite nanotubes, mesenchymal stem cells

## Abstract

Here we report the use of forskolin-modified halloysite nanotubes (HNTs) as a dopant for biopolymer porous hydrogel scaffolds to impart osteoinductive properties. Forskolin is a labdane diterpenoid isolated from the Indian Coleus plant. This small molecule is widely used as a supplement in molecular biology for cell differentiation. It has been reported in some earlier publications that forskolin can activate osteodifferentiation process by cyclic adenosine monophosphate (c-AMP) signalling activation in stem cells. In presented study it was demonstrated that forskolin release from halloysite-doped scaffolds induced the osteodifferentiation of equine mesenchymal stem cells (MSCs) in vitro without addition of any specific growth factors. The reinforcement of mechanical properties of cells and intercellular space during the osteodifferentiation was demonstrated using atomic force microscopy (AFM). These clay-doped scaffolds may find applications to accelerate the regeneration of horse bone defects by inducing the processes of osteodifferentiation of endogenous MSCs.

## 1. Introduction

Mesenchymal stem cells are multipotent somatic stem cells that have been investigated as a treatment for a broad range of diseases such as diabetes [1], myocardial infarction, stroke [2], acute and chronic inflammatory disorders and various autoimmune diseases, etc. [3]. The safety of MSCs transplantation has been proven in various clinical tests both for allogeneic and autologous transplants that allow for creation of an inventory of third-party donor MSCs to use a single isolation for the treatment of numerous patients. [4]. The low immunogenicity and immunomodulatory features [5], easy to perform cryopreservation storage requirements, high potential for in vitro differentiation and self-renewal capacity without any severe abnormalities stimulate the use of MSCs as a powerful tool having an excellent therapeutic potential for regenerative medicine [6]. The International Society for Cellular Therapy has established the minimum criteria for characterizing of MSCs, that is MSCs express cell surface markers such as clusters of differentiation CD29, CD44 CD73, CD90, CD105, and CD 146 but not hematopoietic cells and endothelial cells markers such as CD31, CD 34, CD 45 [7,8] and human leucocyte antigen HLA-DR [3]. Under suitable differentiation cultural medium and conditions, multipotent stem cells have the capacity to differentiate in vitro into mesodermal lineage [5] and give rise to osteoblasts, chondrocytes, adipocytes and myocytes [9] ectodermal (neurocytes) and endodermal lineages (hepatocytes) [5]. Bone marrow MSCs were isolated and investigated for the first time and currently also recognized other origins of such somatic stem cells including adipose tissue, skeletal muscle, amniotic fluid, breast milk, endometrium, dental pulp, placenta, umbilical cord and Wharton’s jelly [10]. Thus, by the beginning of the 21st century, it was concluded that MSCs are found in the connective tissue of many organs. However, it should be noted that MSCs populations from different tissues are not functionally equivalent in terms of differentiation potential, especially in vivo [11]. Adipose tissue as a source of MSCs has several advantages: MSCs derived from adipose tissue (ADSCs) exhibit similar morphology and immune phenotype when compared with bone marrow stem cells (BMSCs) and umbilical cord mesenchymal stem cells (UC-MSCs). The amount of ADSCs in adipose tissue exceeds the amount of BMSCs in Medullary stroma. Through liposuction, it is possible to obtain about 2–6 × 10^8^ cells in 300 mL of aspirate, which is approximately 500 times much than the number of stem cells obtained from bone marrow [12]. Adipose derived MSCs are capable to osteogenic differentiation, the success of which is influenced by many factors (composition of cultural medium, transcription and growth factors, small signalling molecules etc.) and the methodology used for MSCs isolation [12,13]. In practical veterinary medicine, equine MSCs have been shown to be effective in the treatment of diseases of the musculoskeletal system of racehorses, such as tendinopathy, meniscus rupture, and cartilage damage. In addition, MSCs are also used to treat acute inflammatory response, as well as systemic inflammatory response syndrome to chronic diseases characterized by long-term low levels of inflammation, such as equine asthma and recurrent uveitis [14,15].

The cell fate is determined by several factors, including the microenvironment signals during tissue formation. The level of cellular response to a microenvironment is can be restricted firstly by ability to respond and interpret signals and secondly by the variety of them [16]. Mechanical signals of scaffold during evaluation of differentiation potential are often not considered [17]. 

Nanomaterials have also been used to stimulate MSCs proliferation and assist cellular osteogenic differentiation [18]. Nanotechnological approaches suggest generating biomimetic scaffolds for bone tissue engineering as a container with a long-term leakage of growth factors to cells or to alter the biophysical environment. Addition of core–shell-structured zeolitic imidazolate framework-8 nanoparticles with in situ growth of hydroxyapatite to form the nanoparticles shell effectively enhanced the mineralization ability of poly-l-lactic acid (PLLA) scaffolds and promoted cell adhesion, proliferation, and differentiation [19]. In situ grown hydroxyapatite in combination with silver nanoparticles on the PLLA scaffold surface promote the osteogenesis in vivo as well as strong antibacterial activity [20]. The chitosan scaffolds conjugated with gold nanoparticles were investigated as a differentiation inductor of MSCs into osteoblasts, suggesting the osteoinductive activity associated with the mechanical properties of the scaffolds regulated mRNA expression and mineralization by activating the Wnt/β-catenin signalling pathway [21]. Nanofibres from polycaprolactone (PCL) were used to generate a collagen I-chitosan-PCL nanocomposite scaffold. These scaffolds enhanced osteogenic differentiation of bone marrow derived stem cells as evidenced by increased mineralisation and the osteogenic markers synthesis [17]. The nanostructured scaffolds are required to simulate the natural mechanical signals from the natural environment to complete formation of the cell niche. Artificial scaffolds should ideally replicate the complex architectural structure of the native tissue, which, for example, is retained by the natural decellularized matrix [16]. It is also important to preserve the benefits of scaffolds as non-immunogenicity and corresponding mechanical properties for certain extracellular matrix (ECM). 

Forskolin is a unique structurally complex labdane-type triterpenoid from a natural source, the plant *Coleus forskohlii*. Forskolin known as a cyclic AMP booster and has widely been used to promote cellular responses to neurotrophins in cell physiology and as a component of induction medium for differentiation of adipose-derived stem cells into Schwann cell-like cells [22]. Forskolin and cAMP in serum-free media were demonstrated to induce the expression of neuronal-specific markers in bone marrow MSCs [23]. Forskolin also directly activates adenylyl cyclase and can stimulate osteogenic differentiation [24,25,26].

Halloysite represents a promising nanomaterial for the loading and sustained release of broad range of biological active compounds including small molecules [27,28]. Halloysite nanotubes *per se* as well as nanostructured polymeric materials with halloysite as dopant expressed low level of toxicity for different types of cells and organisms [29]. Here we demonstrate the effective osteogenic differentiation of MSCs using ECM-mimicking biopolymer scaffolds doped with forskolin-loaded halloysite nanotubes.

## 2. Materials and Methods

### 2.1. Isolation and Culture of MSCs

Subcutaneous equine adipose tissue was obtained by excision under local anesthesia with lidocaine solution at the area of the tail base of a 1.5-year-old horse. Permission from the local ethics committee of Kazan Federal University was obtained to carry out procedures for the collection of adipose tissue (No. 1 from 23 February 2015, “Genetic and cell therapy in regenerative veterinary medicine”). Samples were transported to the cell culture laboratory and processed within 4 h. The adipose tissue was minced, centrifuged at 2000 rpm for 10 min to remove excessed lipid fraction, and digested using 0.2% collagenase from hepatopancreas of crab with approximate collagenolytic activity about 750 U/mg (Biolot, Saint-Petersburg, Russia) in Hanks’ Balanced Salt Solution (HBSS) with calcium and magnesium (Gibco, Waltham, MA, USA) at 37 °C for 1 h with agitation. The homogenate was centrifuged at 1500 rpm for 5 min and the enzymatic solution was decanted. The cell pellet was suspended with a Dulbecco’s phosphate-buffered saline (DPBS; PanEco, Moscow, Russia) solution and centrifuged to remove residual enzymes. The obtained cells were cultured in Minimal Essential Medium alfa-modification (α-MEM) supplemented with 10% fetal bovine serum (FBS; PAA Laboratories Inc., Ontario, Canada), 100 U/mL penicillin, 100 μg/mL streptomycin, and 2 mM L-glutamine. When the cells had reached 80% of confluence, they were detached from the culture dish using trypsin, suspended in cryopreservation medium containing 10% of dimethyl sulfoxide (DMSO; AppliChem Gmbh, Darmstadt, Germany), frozen and stored in liquid nitrogen for later use. After the monolayer expansion, cells were used for the experiments at passage 3–5. Stemness of resulted cells was confirmed using immunocytochemical staining and flow cytometry analysis of expression of cluster of differentiation (CD29, CD44 CD73, CD90) [30].

### 2.2. Differentiation of MSCs 

The equine adipose derived MSCs were analyzed for their capacity to differentiate into the adipogenic, osteogenic and chondrogenic lineage. Differentiation was performed in monolayer cultures. Cells of the third passage were plated on 12-well plates with round plastic cover slips (30,000 cells per well) and incubated in complete α-MEM growth medium until a monolayer culture was obtained (48 h). Then cells were transferred to a specific osteogenesis, adipogenesis, chondrogenesis differentiation kits MesenCult™ (STEMCELL Technologies, Kent, WA, USA) to induce differentiation in three directions [31]. The media were replaced every 3 d. After 14 d of incubation with differentiating media, the cell cultures were fixed with 4% paraformaldehyde for 20 min at room temperature, and samples for the light microscopy, AFM and dark-field microscopy were prepared. To determine the mineralization (osteogenic differentiation), von Kossa reaction was used [32]. Briefly, silver nitrate solution (2% (*w*/*v*)) was applied on the cells with subsequent incubation for 1 h under bright illumination. Combination of light and dark field microscopy was used to visualize the oil droplets in the cells, which were incubated in adipogenic differentiation medium. Mucopolysaccharides specific stain was used to detect chondrogenic differentiation the staining for mucopolysaccharides was used. Briefly, the fixed cells were washed with phosphate buffer saline three times for 5 min, stained for 1 h with Alcian Blue (1 g Alcian Blue/100 mL 0.1 M HCl) and then washed with DPBS. During the differentiation process, cultures were monitored intravitally once per 24 h using an inverted AxioObserver A1 microscope (Carl Zeiss, Oberkochen, Germany) in phase contrast mode. The images were obtained using an AxioImager microscope (Carl Zeiss, Oberkochen, Germany) and processed with ZEN software 2.0 (blue edition).

### 2.3. Forskolin-HNTs Fabrication

A total of 10 mg of Stemolecule™ Forskolin (Reprocell, Beltsville, MD, USA) were dissolved in DMSO to obtain a 10× stock solution. Then 10× stock solution was diluted with deionized water to obtain a working solution with a concentration of 10 mM. 200 µL of 10 mM working solution of Forskolin was mixed with dry HNTs (30 mg) in centrifuge tube, suspended on vortex mixer for 10 min and placed into desiccator for loading by vacuum, as described previously [33]. Forskolin loading procedure was performed for 24 h. The loading efficiency was evaluated by Fourier transform infrared spectroscopy (FT-IR).

### 2.4. Fourier Transform Infrared Spectroscopy

Fourier Transform Infrared Spectroscopy (FT-IR) HNTs, forskolin and forskolin-loaded HNTs were registered using a Frontier FT-IR spectrometer FT-801 (OSTEC FTIR Spectrometer, Russia). The measurements were recorded at room temperature in the range between 500 and 4000 cm^−1^ with a spectral resolution of 2 cm^−1^. 

### 2.5. Osteogenic Differentiation of MSCs on Polymeric Nanostructured Scaffolds

The biopolymeric scaffolds composed from gelatin, chitosan and agarose with pure HNTs (control scaffold) and forskolin-loaded HNTs (6 wt.%) were obtained via a freeze-drying technique as described previously [34]. Briefly, 1% pure gold gelatin 180 bloom (Roth, Karlsruhe, Germany), 1% medium molecular weight chitosan (Sigma-Aldrich, St. Louis, MO, USA) and 2% agarose, high melting temperature, medium resolution (Alfa Aesar, Ward Hill, MA, USA) were dissolved in 1% of acetic acid with pure HNTs or forskolin-loaded HNTs (6 wt.%) and incubated at 80 °C for 2 h in water bath with magnetic stirrer. Then the gel was casted into molds with appropriate shape and frozen at −80 °C for 24 h followed by the freeze-drying procedure at −80 °C. A column of 2 cm in diameter and 1.5 cm in height was formed and then sliced (thickness ~3 mm) for the further use. Sliced scaffolds were introduced to UV irradiation for 2 h for the sterilization. Scaffolds were placed in 12 well plates and equilibrated in complete α-MEM overnight. Further, media were aspirated and equine MSCs (2 × 10^6^ cells in 2 mL of α-MEM per well) were applied onto the sliced scaffold and incubated for 14 d with every 48-h medium change. After that, samples were washed with DPBS and fixed with 4% paraformaldehyde for 20 min at room temperature. The efficacy of osteogenic differentiation under influence of forskolin without the differentiation medium was estimated using von Kossa staining (Section 2.2) and microscopy analysis using an AxioScope A1 microscope (Carl Zeiss, Oberkochen, Germany). Osteogenic differentiation in parallel was performed on 2D scaffolds with the same composition for the AFM visualization. Then, 2D scaffolds were obtained as a thin film from biopolymeric solution doped with pure HNTs, forskolin-loaded HNTs (6 wt.%) or from pure polymeric solution on cover slips (15 mm Ø) placed into 12 well plates. After distribution of polymeric solution, cover slips were placed under UV irradiation for 2 h for the sterilization and polymerization. Further processing was the same as described for the 3D scaffolds. MSC were applied on 2D in the amount of 20,000 cells in 2 mL of α-MEM per well. 

### 2.6. Dark-Field Microscopy

Dark-field microscopy images of MSCs after 14 d differentiation process were obtained using an Olympus BX51 upright microscope equipped with a CytoViva^®^ enhanced dark-field condenser, a fluorite 100× objective and DAGE CCD camera [35]. Cells were grown on plastic adhesive cover slips without scaffold in appropriate differentiating media media (α-MEM was used as a control). After 14 d, cells were washed twice with DPBS, fixed with 4% paraformaldehyde for 20 min in room temperature with subsequent extensive washing and staining of nuclei with DAPI for cellular nuclei visualization and embedded in Moviol mounting media. An X-cite 120Q wide-field fluorescence microscope excitation light source (Excelitas Technologies, Vaudreuil-Dorion, QC, Canada) and CytoViva^®^ Dual Mode Fluorescence system (CytoViva, Inc., Auburn, AL, USA) equipped with a Triple Pass Filter were used to image DAPI-stained cellular nuclei in transmitted fluorescence illumination mode; the exposure time was set at 100 µs.

### 2.7. Atomic Force Microscopy (AFM) 

For AFM cells were seeded onto cover slips with 2D scaffold film (Section 2.5) and cultured for 14 d. After that samples were extensively washed with deionized water and dried on air at room temperature. AFM images were obtained using a Dimension Icon microscope (Bruker, Billerica, MA, USA) operating in PeakForce Tapping mode. Cells were imaged using ScanAsyst-Air probes (Bruker, Billerica, MA, USA) (nominal length 115 μm, tip radius 2 nm, spring constant 0.4 Nm^−1^). The images were obtained at 512 lines/scan at 0.8–0.9 Hz scan rate and up to 1 nN peak force setpoint. Topography was visualized in height sensor and peak force error channels. The AFM data were processed using Nanoscope Analysis v.1.7 software (Bruker, Billerica, MA, USA) [36]. The Derjaguin–Mueller–Toporov (DMT) model was chosen to find the Young’s modulus of a surface to determine mechanical characteristics. This model refines the simplest model (Hertz), taking into account adhesion outside the contact area, and is more applicable to small cantilevers (Heinz and; Hoh, 1999) which we used here.

### 2.8. 3D laser Scanning Confocal Microscopy

Surface topography imaging and characterization was performed using a VK-X150 3D laser scanning confocal microscope (Keyence, Osaka, Japan) equipped with a 20× Nikon fluorite objective. The images were obtained in “superfine” mode, collecting simultaneously optical multifocus reconstruction, laser intensity and height data. The specimens were placed onto regular microscopy glass slides and imaged in air under ambient conditions. MultifileAnalyser v. 1.3.0.116 software (Keyence, Osaka, Japan) was used to process the data. For surface roughness numerical characterization, the flat areas on the images the selected.

## 3. Results

**Figure 1:**
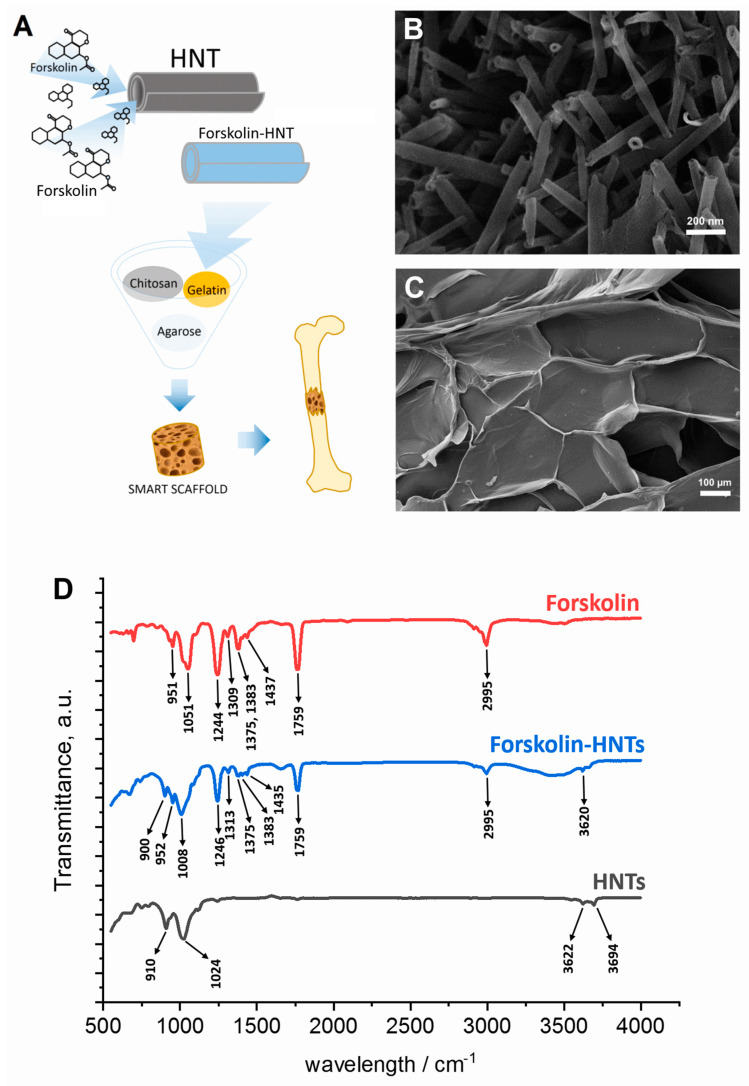
(**A**) Scheme demonstrating the forskolin-doped polymer scaffold fabrication (**A**) and its characteristics: (**B**,**C**)—SEM images of HNTs and scaffold surface, respectively, (**D**)—FT-IR spectrum of forskolin loaded HNTs. The scale bars in (**B**,**C**) are 200 nm and 100 μm, respectively.

Figure 1 shows the scheme of smart scaffold with sustained release of forskolin fabricating (Figure 1A). SEM images demonstrate the tubular structure of halloysite nanotubes with characteristic inner lumen which can be loaded by wide range of functional compounds (Figure 1B) and surface of 3D porous biopolymeric scaffold obtained by freeze-drying technique (Figure 1C). The FT-IR analysis was further undertaken to identify the major functional groups involved in the binding of forskolin into HNTs. The FT-IR spectra of forskolin-HNTs corresponded to the forskolin spectra in the 950–1800 cm^−1^ wavenumbers region. The area of 3600–3700 cm^−1^ forskolin-HNTs corresponded to HNTs. The wavelength range between 950 cm^−1^ and 1800 cm^−1^ can be considered as a fingerprint region for forskolin, which presents numerous characteristic signals many of them detected only for forskolin-HNTs and not detected for HNTs. The typical FT-IR peaks of the forskolin at 1375 and 1383 cm^−1^ (bending of CH_3_) were represented by less intense signals in the case of the forskolin-HNTs.

**Figure 2:**
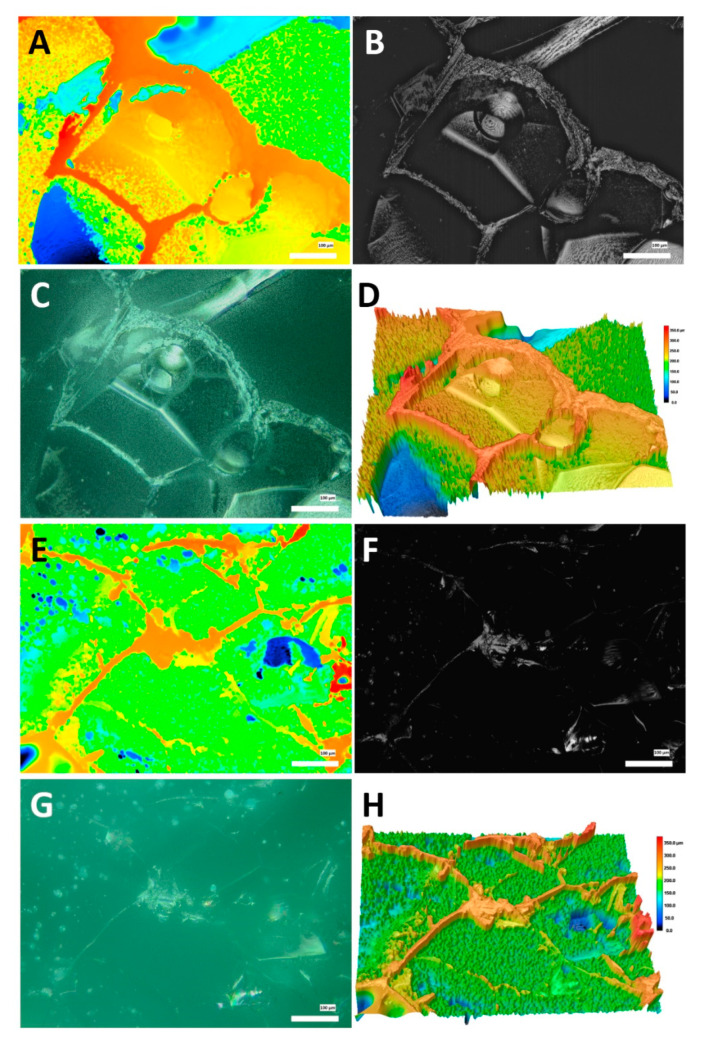
Surface topography of 3D porous scaffold visualization in different modes. (**A**–**D**)—scaffold without HNTs, (**A**)—height mode, (**B**)—laser intensity mode, (**C**)—optical micrograph, (**D**)—3D height rendering mode; (**E**–**H**)—scaffold doped with HNTs, (**E**)—height mode, (**F**)—laser intensity mode, (**G**)—optical micrograph, (**H**)—3D height rendering mode.

The surfaces of 3D polymer scaffolds were visualized and quantitatively characterized using 3D laser scanning microscopy, the typical optical, laser intensity and height (2D and 3D) images are given in Figure 2. 

In both cases, the surface structure of the scaffolds is sponge-like, with crater-like features, peaks and valleys along with relatively flat areas. The selected surface topography parameters measured are summarized in Table 1. We found that doping the scaffolds with halloysite manifests in reduction of surface roughness (root mean square height, Sq) and smoothening of the surfaces (reduced valley height, Svk), whereas the kurtosis (Sku) value was unchanged. In addition, the reduction skewness (Ssk) in halloysite-doped scaffolds indicated that the features height difference in the scaffolds was distributed uniformly, whereas in the halloysite-free scaffolds the surfaces features were distributed to the lower side of the structure. These results suggest that halloysite as a filler of the polymer scaffolds increases the overall volume of the composite and fills the voids in the polymer structure. As a result, the cells’ attachment is facilitated since the surfaces becomes more suitable for cellular spreading and proliferation.

**Figure 3:**
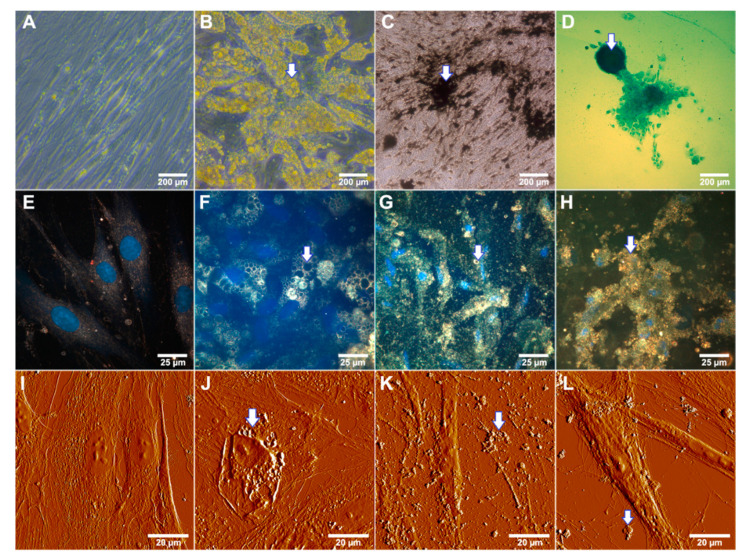
Differentiation potential of equine adipose derived mesenchymal stem cells after 14 d incubation in differentiating media. Arrows indicate characteristic changes of cellular morphology. (**A**)—cells incubated in the presence of α-MEM (negative control); (**B**)—cells grown under adipogenic conditions (note a huge amount of yellow colored lipid droplets); (**C**)—von Kossa staining of cells differentiated to the osteoblasts under osteogenic conditions (note the black stained calcium accretion); (**D**)—alcian blue staining of cells differentiated to the chondrocyte lineage in chondrogenic medium (polyanionic glycosaminoglycan chains of proteoglycans visualized as a blue agglomerate). (**E**–**L**)—Label-free visualization of differentiated MSCs under specific conditions, (**E**–**H**)—dark-field microscopy; (**E**)—control (α-MEM), (**F**)—adipogenic differentiation, (**G**)—osteogenic differentiation, (**H**)—chondrogenic differentiation; nuclei were stained with DAPI and detected using fluorescence mode; (**I**–**L**)—AFM detection; (**I**)—control, (**J**)—adipogenic differentiation, (**K**)—osteogenic differentiation, (**L**)—chondrogenic differentiation.

Specific staining of differentiated cells allowed us to confirm the ability of adipose derived cells develop into multiple specialized cell types. We confirmed that the equine adipose derived MSCs used in this study could be differentiated in three directions: adipogenic, osteogenic and chondrogenic using the commercially available media with specific factors and supplements which induce the morphological and functional changes (Figure 3). Osteogenic conditions lead to the mineral matrix formation which can be visualized as black extracellular structures using the von Kossa reaction with silver nitrate (Figure 3C) as well as in dark-field micrographs as highly refractive inclusions (Figure 3G) and in AFM image as nodules lying on and around the cells (Figure 3K). MSC grown in adipogenic media exhibits extensive intracellular lipid vacuoles formation typical of mature adipocytes after 14 d of cultivation (Figure 3B,F,J). Chondrogenic differentiation promotes the formation of an extracellular matrix based on glycosaminoglycan visibly as round shaped agglomerates blue stained with Alcian Blue (Figure 3D).

We have demonstrated the characteristic changes in cellular morphology and appearance of intracellular and extracellular inclusions under osteogenic, chondrogenic and adipogenic conditions using enhanced dark-field microscopy and AFM as label-free technique. Control cells grown in α-MEM exhibited the fibroblast-like morphology characteristic of MSCs (Figure 3A,E,I). Lipid droplets using these techniques visualized more pronounced (Figure 3F,J) as well as the agglomerates of calcium salts in case of osteogenic differentiation (Figure 3G,K). During the osteogenic differentiation cells decreased in size, the same effect can be observed in the case of chondrogenic differentiation on dark-field micrograph (Figure 3H). AFM imaging of cells in the process of chondrogenic differentiation allowed visualizing the extracellular inclusions possibly composed of glycosaminoglycan (Figure 3L). 

**Figure 4:**
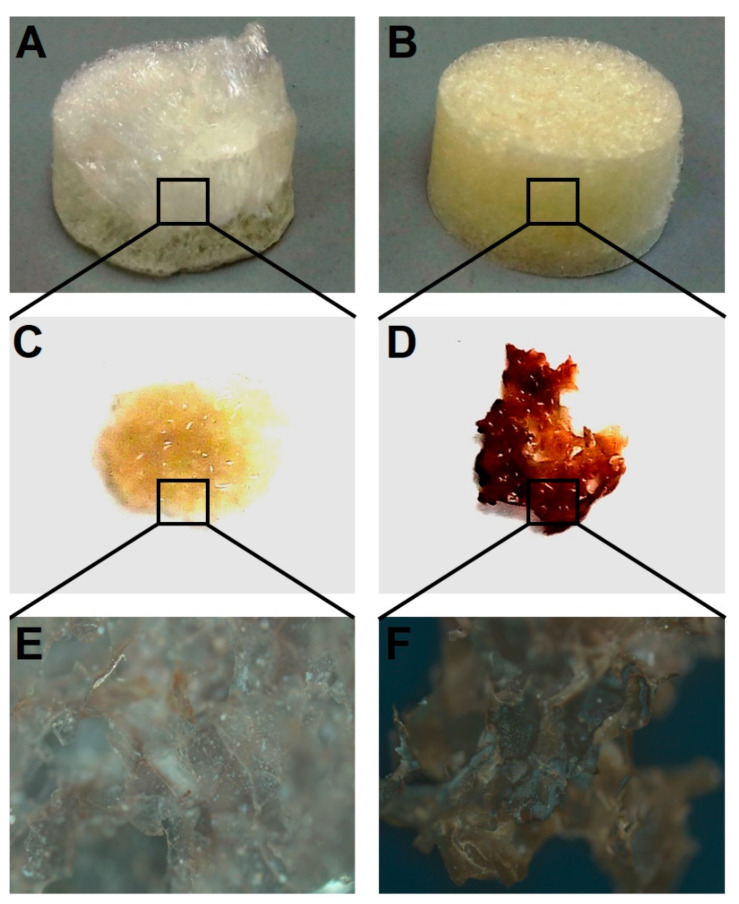
Qualitative analysis of osteogenic differentiation of MSCs on chitosan–agarose–gelatin scaffolds in presence of forskolin-loaded HNTs. Macroscopic view of scaffold with pristine HNTs (**A**) and scaffold doped with forskolin-loaded HNTs (**B**) before MSCs seeding; von Kossa staining of fragment of control scaffold with pristine HNTs (**C**) and scaffold doped with forskolin-loaded HNTs (**D**) with seeded MSCs after 14 d of incubation; visualization of control scaffolds with pristine HNTs (**E**) and scaffold doped with forskolin-loaded HNTs (**F**) with seeded MSCs after 14 d of incubation.

Next, we performed the macroscopic qualitative analysis of the possibility of MSCs differentiation into osteogenic lineage on 3D porous scaffolds doped with forskolin (Figure 4). Using simple and rapid technique of von Kossa staining we have demonstrated the possibility of MSCs grown on 3D polymeric scaffolds to transform into osteoblasts without specific media. 

**Figure 5:**
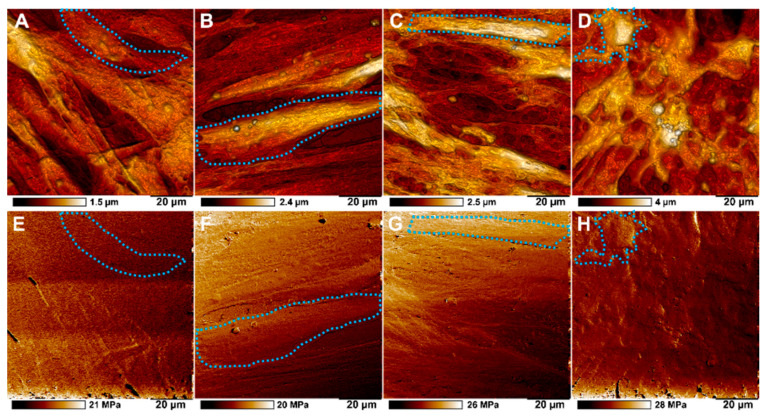
AFM imaging of topography (upper row) and adhesion (lower row) of MSCs during osteogenic differentiation on biopolymer scaffolds. (**A**,**E**)—MSCs grown on control scaffold without HNTs in α-MEM, (**B**,**F**)—MSCs grown on scaffold with pristine HNTs in α-MEM; (**C**,**G**)—MSCs grown on control scaffold with pristine HNTs under osteogenic conditions, (**D**,**H**)—MSCs grown on scaffold with forskolin-loaded HNTs in α-MEM. Single cells are marked with blue dotted line.

The samples of cells differentiated on a biopolymer nanostructured matrix were studied using atomic force microscopy (Figure 4). We found that the introduction of halloysite nanotubes containing forskolin into a biopolymer hydrogel significantly affects the process of osteodifferentiation of MSCs. On scaffolds that did not contain modified nanotubes, the cells exhibited a characteristic MSC morphology (Figure 5A,B,E,F), whereas on scaffolds doped with forskolin nanotubes, clear changes in cell morphology and spherical 3D nodules formation were observed (Figure 5D,H). These changes were more pronounced than in case of MSCs incubation in osteogenic medium on scaffolds with pure HNTs (Figure 5C,G).

Cells cultured on matrices doped with forskolin loaded nanotubes exhibited slight enhancement in stiffness in comparison with cells grown on scaffolds without nanotubes as well as on scaffolds with pristine HNTs. The transformed MSCs monolayer showed comparable nonspecific adhesion with MSCs grown on matrices with pristine nanotubes under osteogenic conditions of specific cultural medium as well as on matrices with HNTs in α-MEM (Table 2).

## 4. Discussion

Bone regeneration in vivo proceeds more successfully in the presence of a large pool of MSCs, which are capable of differentiating into a cartilaginous mass in the region of the vessels of damaged tissues with its subsequent ossification [37]. In previous studies the successful isolation, the multipotential differentiation capacity as well as the self-renewing potential of MSCs have also been described in the horse [38]. The cells used in our study were isolated for the clinical use in veterinary traumatology. To maintain high titers of stem cells in the area of bone tissue defect, it is promising to add them on three-dimensional biopolymer carriers, the composition and mechanical properties of which can be modulated by using various types of polymers, dopants, including nanomaterials, and by methods of matrix formation. At present, different types of matrices have been developed based on various polymers, both artificial and natural, suitable for transplantation. A promising approach to matrix formation is the addition of nanomaterials to polymer composition. The nanocomposite matrices obtained in this way, while retaining all the advantages of polymer materials, acquire new properties that can increase their biocompatibility, functionality and, thus, expand the scope of their application [39]. However, works in this direction do not lose their relevance. To successfully solve the problems of regenerative surgery, matrices for transplantation must meet a number of requirements, in particular, have a low immunogenicity, a high degree of biocompatibility and biodegradability, as well as promote the proliferation of both own endogenous stem cells and introduced ones. In the study of osteogenic differentiation of MSCs, the role of mechanical signals from the scaffold remains poorly understood [40]. Previously we have obtained and characterized tissue engineering scaffolds based on biopolymers (gelatin, agarose, chitosan), doped with nanotubes of the mineral halloysite [34]. Nanomodification made it possible to achieve a significant increase in the mechanical strength of the material when adding only 6 wt.% of nanotubes during the formation of a porous hydrogel obtained by lyophilization. Porous matrices obtained in this way had the ability to support the growth and proliferation of various types of human cancer lines (A549—human lung carcinoma cells, Hep3B—human hepatoma cells, PC3—human prostate carcinoma cells). Nanomodified matrices were implanted subcutaneously into rats with complete resorption of the material and restoration of blood supply within 6 weeks, which proved their safety and potential for use in clinical practice. Such a material with replacement of native HNTs with forskolin-loaded ones was used in presented study. 

Numerous studies have proven the biosafety of halloysite nanotubes for various organisms [28,41,42,43]. In addition, this material has unique properties—the lumen of nanotubes can be loaded with various compounds, including drugs, as well as growth factors that was demonstrated in our previous studies [33,44,45,46]. Loading of functional substance into nanotubes facilitates its sustained release, as shown in our previous study [47]. In this study, we used forskolin-loaded halloysite nanotubes for nanomodification of a biopolymer scaffold, which provided an even more gradual release of the functional substance. 

Loading efficacy of forskolin in HNTs was estimated by FT-IR and showed effective binding of nanotubes and forskolin based on the obtained spectra combining the fingerprint region for forskolin and halloysite. The broadening of the absorption band in the region of 3000–3500 cm^−1^ may indicate the appearance of hydrogen bonds between forskolin and the internal surface of HNTs consists of a gibbsite-like array of Al-OH groups [48].

Molecular mechanism of bone tissue fabrication could be affected by specific factors, which can initiate and promote the osteogenesis. It has been shown that protein kinase A (PKA) activation forms an important component of the mechanism by which BMP-2 mediates MSC differentiation. After activation PKA phosphorylates its prototypical downstream transcription factor, cAMP-responsive element binding protein (CREB), which is associated with a number of important physiological functions including osteochondrogenic differentiation [49]. Here we demonstrated the osteogenic differentiation of MSCs on the biopolymeric nanostructured scaffolds with forskolin-loaded HNTs. In this system HNTs were used as nanocontainers for the slow release of forskolin, which is a known as stimulator of bone morphogenic protein-2 (BMP-2) formation by activating cAMP synthesis [26].

We applied high-resolution microscopy techniques (AFM and enhanced dark-field microscopy) for the label-free visualization of morphological and structural changes of cells which arise in the process of MSC differentiation. Recently, there has been an increase in interest in this technology to distinguish between different morphotypes of cells and their functional state [50]. AFM and dark-field microscopy allowed us to reveal the differences in cells and ECM after their three-lineage differentiation. In addition, the AFM method revealed mechanical changes in the MSC monolayer after osteogenic differentiation. We suppose that higher rigidity of transformed cells observed due to nodules formed from calcium salts on the cell surface and in the intercellular space, which were detected by staining of mineral matrix formed on biopolimeric 3D scaffold with forskolin-loaded nanotubes during MSCs growth on the surface of scaffold and differentiation. These observations confirm the process of osteodifferentiation under the influence of nanomodification of a biopolymer hydrogel with forskolin-loaded nanotubes.

## 5. Conclusions

In our study, we demonstrated the stemness of equine adipose derived cells and their multipotency which expressed in possibility of cells to differentiate into discrete cell types and confirms that cells belong to MSCs. Modification of nanotubes with forskolin, a compound belonging to the group of so-called small molecules, made it possible to obtain a new osteoconductive smart polymeric scaffold. This material promoted osteodifferentiation of horse adipose tissue derived mesenchymal stem cells without addition of specific osteogenic media. Label free techniques of visualization have been successfully applied to confirm the changes occurring in cells during osteodifferentiation. We suggest that synergistic action of the mechanical properties of the substrate and the directed chemical signal from forskolin most likely promotes differentiation of MSCs comparable in time and efficiency with the conditions provided by the differentiating media. At the same time, further detailed confirmation of this phenomenon is required, including on other types of cells using methods of enzymatic and analysis of gene induction, which are marker for the process of osteodifferentiation.

## Figures and Tables

**Table 1:** Roughness parameters in polymer scaffolds as measured using 3D confocal laser scanning microscopy.

Specimen	Sq/µm	Ssk	Sku	Svk/µm
HNTs-free scaffolds	27.1 ± 9.1	0.35 ± 0.4	3.12 ± 0.5	19.9 ± 4.1
HNTs-doped scaffolds	13.4 ± 1.4	−0.04 ± 0.1	3.23 ± 0.5	12.4 ± 0.9

**Table 2:** Mechanical characteristics of MSC monolayer (scan area 30 × 30 µm) of cells after cultivation under osteogenic (osteo) and non-osteogenic conditions (control).

Control − HNT		Control + HNT		Osteo + HNT		Forskolin + HNT	
**Adhesion** **nN**	**Modulus** **MPa**	**Adhesion** **nN**	**Modulus** **MPa**	**Adhesion** **nN**	**Modulus** **MPa**	**Adhesion** **nN**	**Modulus** **MPa**
3.2 ± 0.4	24.6 ± 3.2	3.9 ± 0.6	25.1 ± 5.1	5.7 ± 3.1	26.8 ± 3.6	4.7 ± 0.8	31.4 ± 3.6
